# A rat model of adenoid hypertrophy constructed by using ovalbumin and lipopolysaccharides to induce allergy, chronic inflammation, and chronic intermittent hypoxia

**DOI:** 10.1002/ame2.12396

**Published:** 2024-04-04

**Authors:** Anqi Liu, Yixing Zhang, Yan Lin, Xuejun Li, Shuming Wang, Wenyan Pu, Xiuxiu Liu, Zhiyan Jiang, Zhen Xiao

**Affiliations:** ^1^ Department of Pediatrics Longhua Hospital Affiliated to Shanghai University of Traditional Chinese Medicine Shanghai China; ^2^ Department of Pediatrics Lishui Hospital of Traditional Chinese Medicine Lishui China

**Keywords:** allergic rhinitis, hypoxia, nasopharynx‐associated lymphoid tissue, rat model of adenoid hypertrophy, upper respiratory inflammation

## Abstract

**Background:**

Adenoid hypertrophy (AH) is a common pediatric disease that significantly impacts the growth and quality of life of children. However, there is no replicable and valid model for AH.

**Methods:**

An AH rat model was developed via comprehensive allergic sensitization, chronic inflammation induction, and chronic intermittent hypoxia (CIH). The modeling process involved three steps: female Sprague–Dawley rats (aged 4–5 weeks) were used for modeling. Allergen sensitization was induced via intraperitoneal administration and intranasal provocation using ovalbumin (OVA); chronic nasal inflammation was induced through intranasal lipopolysaccharide (LPS) administration for sustained nasal irritation; CIH akin to obstructive sleep apnea/hypopnea syndrome was induced using an animal hypoxia chamber. Postmodel establishment, behaviors, and histological changes in nasopharynx‐associated lymphoid tissue (NALT) and nasal mucosa were assessed. Arterial blood gas analysis and quantification of serum and tissue levels of (interleukin) IL‐4 and IL‐13, OVA‐specific immunoglobulin E (sIgE), eosinophil cationic protein (ECP), tumor necrosis factor (TNF‐α), IL‐17, and transforming growth factor (TGF)‐β were conducted for assessment. The treatment group received a combination of mometasone furoate and montelukast sodium for a week and then was evaluated.

**Results:**

Rats exhibited notable nasal symptoms and hypoxia after modeling. Histopathological analysis revealed NALT follicle hypertrophy and nasal mucosa inflammatory cell infiltration. Elevated IL‐4, IL‐13, IL‐17, OVA‐sIgE, ECP, and TNF‐α levels and reduced TGF‐β levels were observed in the serum and tissue of model‐group rats. After a week of treatment, the treatment group exhibited symptom and inflammatory factor improvement.

**Conclusion:**

The model effectively simulates AH symptoms and pathological changes. But it should be further validated for genetic, immunological, and hormonal backgrounds in the currently used and other strains and species.

## Graphical abstract

An AH rat model was created via comprehensive allergic sensitization, chronic inflammation induction, and chronic intermittent hypoxia. By using NALT as an analogy to adenoids, we observed reactive lymphoid follicle hyperplasia in rat NALT after modeling, with pathological changes resembling enlarged adenoids and tonsils.

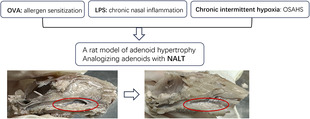

## 1 INTRODUCTION

Adenoid hypertrophy (AH) is a common respiratory disease in children and a significant cause of pediatric obstructive sleep apnea/hypopnea syndrome (OSAHS), which can lead to otitis media,^1^ sinusitis,^2^, ^3^ behavioral disorders,^4^, ^5^ and even impaired cardiopulmonary function,^6^ severely affecting the growth, development, and quality of life. Adenoidectomy is considered the primary treatment for AH.^7^ However, the impact of adenoidectomy on immune function remains controversial.^8^, ^9^ There is also a certain recurrence rate after surgery.^10^ On the contrary, medications for AH are limited to corticosteroids and leukotriene receptor antagonists,^11^ which have an unsatisfactory efficacy and a tendency to relapse. Therefore, seeking safe and effective drug therapies is urgently needed in pediatric clinics. New drug development requires rigorous preclinical and clinical studies, and preclinical research is inseparable from the establishment of animal models. However, currently no translational animal model of AH is available. In this study, we focused on the risk factors of AH and combined allergy, chronic inflammation, and intermittent hypoxia to develop a rat model of AH using rat nasopharynx‐associated lymphoid tissue (NALT) to mimic adenoid tissue.

Together with diffuse lymph nodes within the nasopharyngeal mucosa, the adenoids form part of the NALT system, serving as the primary site of contact with respiratory and digestive antigens. The adenoids are covered by pseudocomplex ciliated columnar epithelium. Similar to other sites of NALT, the adenoid epithelium contains a specialized antigen‐presenting cell, the M cell (microfold cell), which transports antigens across the mucosa and initiates an immune response.^12^, ^13^ In rats and mice, NALT is a pair of lymphoid tissues located in the nasal cavity, which share a very similar structure with adenoids, characterized by a ciliated columnar epithelium with M cells and B‐lymphoid follicles, and they also contribute to mucosal immune responses in the upper respiratory tract. Koornstra et al. initiated a series of studies on the NALT and proposed the concept of Waldeyer‘s ring (WR) equivalent.^14^, ^15^ They found that NALT does not have numerous large crypts or a distinct lymphoreticular epithelium and in this respect resemble the human pharyngeal tonsil more than the palatine tonsil. Therefore, in this study, we used rat NALT to mimic adenoid tissue, hoping to observe AH‐like pathological changes in lymphoid follicular hyperplasia.^16^

The cause of AH in children is not fully understood. It is associated with recurrent infections, allergies, and genetics. Typically, there is a delicate balance between the natural flora of the adenoid and immune responses. However, this equilibrium can be disrupted by recurrent viral and bacterial infections, as well as colonization by pathogens. Frequent infections or allergies in the upper respiratory tract often lead to hypertrophy.^17^ At the same time, the ciliated columnar epithelium of adenoids with M cells may be reduced and chemotaxis to a complex squamous epithelium,^18^, ^19^ which implies that the immune function of the adenoids is weakened. Altered production of cytokines is described in immune disturbances. For example, elevated levels of proinflammatory cytokines such as high‐sensitivity C‐reactive protein, tumor necrosis factor (TNF‐α), IL‐1 (interleukin), and IL‐10 as well as intercellular adhesion molecule‐1 in children with AH have been observed.^20^

Many epidemiological surveys have suggested a strong correlation between allergic rhinitis (AR) and AH.^21^, ^22^, ^23^ As the first mucosal immune tissue encountering allergens, the adenoid participates in the allergic response. The Th17/Treg balance is attributed to both AR and AH. Th17 cells are an important class of effector T cells that regulate the body's fight against extracellular pathogens while also promoting the occurrence of autoimmune and inflammatory diseases. In contrast, human regulatory T cells (Treg) maintain peripheral tolerance. They exert opposite effects on autoimmunity and inflammation. Kobi Sade found a significant negative linear correlation between the Th17/Treg ratio and the patients' clinical scores. Children with AH exhibited a significant increase in the number of Th17 cells and Th17‐related cytokine secretion (IL‐17), and a decrease in the number of Treg cells, Treg‐related cytokine secretion (IL‐10, TGF‐β [transforming growth factor]) levels.^24^ Chenchen Ye found that CCL20/CCR6‐mediated macrophage polarization into M1 type can stimulate the differentiation of AH lymphocytes into Th17 cells.^25^ Similar results were obtained in the other two studies as well.^26^, ^27^ Additionally, increased eosinophils and T‐helper 2 inflammation can manifest in the adenoid. IL‐4, IL‐8, IL‐18, IL‐33, H2R, LTR1, LTR2, and GCR increased in adenoid tissue.^28^ NALT in rodents has also been associated with AR.^29^ Some studies indicate that eosinophil counts in the adenoid and tonsils reflect the severity of AR.^30^, ^31^ Leukotriene receptor antagonists are currently the first‐line drugs for AH.^11^

The chronic intermittent hypoxia (CIH) resulting from sleep‐disordered breathing is implicated in the occurrence and progression of adenoid inflammation.. Hypoxia‐induced local inflammation has been validated in many tissues and diseases, such as sinusitis.^32^ In our previous study, we found that hypoxia upregulates proinflammatory cytokines and cell permeability in human adenotonsillar epithelial cells by activating the SUMO‐1‐HIF‐1α signaling pathway, thereby augmenting local inflammation of the adenoid/tonsil.^33^ Because rat NALT does not appear to undergo hypertrophic proliferation leading to nasal obstruction, we could only artificially induce intermittent hypoxia to mimic OSAHS symptoms.

Given that AH is a multifactorial disease, we aimed to develop a rat model of AH by simulating the risk factors associated with AH, using ovalbumin (OVA) and LPS to induce allergy, chronic inflammation, and CIH.

## 2 MATERIALS AND METHODS

### 2.1 Animals

**FIGURE 1:**
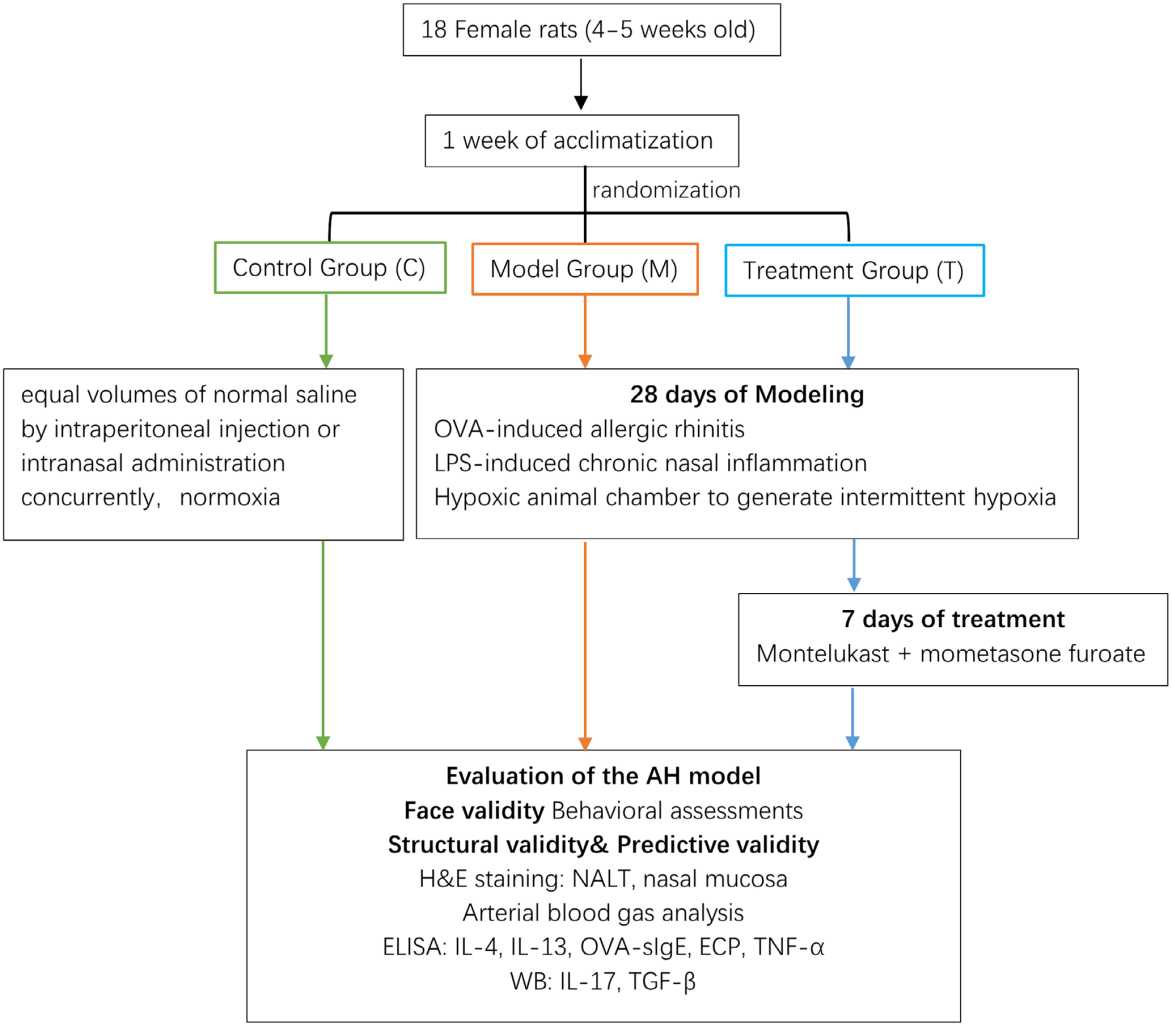
Experimental protocol.

Eighteen female Sprague–Dawley rats (aged 4–5 weeks) were allowed to acclimate for 1 week with free access to food and water in a closed environment at a temperature of 23 ± 3°C, a relative humidity of 55 ± 15%, and a 12‐h light–dark cycle. The animals were fed and observed at the Shanghai University of Medicine and Health Sciences. The procedures were approved by the Research Ethics Committee of Shanghai University of Medicine and Health Sciences (2021‐GZR‐18‐340 406 198 707 142 817) and complied with ethical standards and international conventions on animal experimentation. All efforts were made to maximally reduce animal suffering (Figure 1).

After 1 week of acclimatization, the rats were randomly divided into three groups (six rats per group), control (C), model (M), and treatment (T) groups, using random number generation using SPSS 26.0.

### 2.2 Reagents

OVA (Sigma, E6337‐100 g), aluminum hydroxide powder (Nanjing Chemical Reagent Co., Ltd, C0151520323), LPS (Merck, L2630: 25 mg), montelukast sodium tablets (Lunan Pharmaceutical, State Food and Drug Administration approval no.: H20083372), mometasone furoate aqueous nasal spray (MSD Belgium BVBA/SPRL, registration no.: H20130182), ELISA kit for IL‐4 (Boyan Biotechnology Co., Ltd, BY‐ER300217), ELISA kit for IL‐13 (Boyan Biotechnology Co., Ltd, BY‐ER330199), ELISA kit for OVA‐sIgE (Boyan Biotechnology Co., Ltd, BY‐ER330618), ELISA kit for eosinophil cationic protein (ECP) (Boyan Biotechnology Co., Ltd, BY‐ER330767), and ELISA kit for TNF‐α (Boyan Biotechnology Co., Ltd, BY‐ER330767) were obtained.

### 2.3 Construction of the AH model

#### 2.3.1 OVA‐induced AR

Rats in the model and treatment groups were sensitized by intraperitoneal injection of 0.3 mg of OVA with 30 mg of aluminum hydroxide powder in 1 mL of normal saline every day, seven times in total. Starting from day 15 post‐OVA injection, the rats were held in a head‐down position and given 50 μL of a 50‐mg/mL OVA solution (3 mg of OVA in 100 μL of saline) intranasally using a micropipette in each nostril as a challenge daily for 7 days. From day 22, 50 μL of a 10‐mg/mL OVA solution (1 mg of OVA in 100 μL of saline) was administered intranasally in each nostril daily for 7 days as maintenance^34^; control rats received equal volumes of normal saline by intraperitoneal injection or intranasal administration concurrently.

#### 2.3.2 LPS‐induced chronic nasal inflammation

Starting from day 1 post‐first OVA injection, rats in the model and treatment groups were given 50 μL of 0.2‐mg/mL LPS solution (10 μg of LPS in 100 μL of saline) intranasally every day using a micropipette, 14 times in total, during the OVA challenge and maintenance periods. LPS was administered 2 h in addition to OVA.^34^ Control rats concurrently received equal volumes of normal saline intranasally.

#### 2.3.3 Hypoxic animal chamber to induce intermittent hypoxia

Starting from day 1 post‐first OVA injection, rats in the model and treatment groups were placed in a hypoxic chamber for 2 h daily. The oxygen concentration was set to 3% during this time; the chamber was opened after the oxygen concentration reached 3% for 40 s to restore the oxygen level to 21%. This cycle was repeated to induce intermittent hypoxia for 28 days. Control rats were kept under normoxic conditions throughout.

### 2.4 Evaluation of the AH model

#### 2.4.1 Face validity

Behavioral assessments were performed within 1 h after the final OVA challenge to evaluate nasal symptoms. Scratching, sneezing, and rhinorrhea were scored by summation as follows: scratching 1–5 times: 1 point, 6–10 times: 2 points, and ≥11 times: 3 points; sneezing 1–3 times: 1 point, 4–10 times: 2 points, and ≥11 times: 3 points; and rhinorrhea—to anterior nostril: 1 point, beyond anterior nostril: 2 points, and covering entire face: 3 points.^35^

Rats were observed for tachypnea, cyanosis of lips and nails, restlessness, or somnolence during hypoxic stimulation.

#### 2.4.2 Structural validity

Rats were anesthetized using an intraperitoneal injection of sodium pentobarbital and euthanized by exsanguination via the abdominal aorta. After the skin and muscles of the head were removed, the rat skull was dissected along the midsagittal plane on ice. Part of NALT, located in the nasopharyngeal cavity, flanking the nasal septum, was immediately collected and frozen, whereas another portion was fixed, immersed in Harrison's fixative (absolute ethanol: glacial acetic acid:40% formaldehyde: physiological saline, 40:5:10:45, v/v) through the nasopharyngeal opening in the palate to preserve the nasal cavity tissues.^19^, ^36^ The entire skull was then immersed in Harrison's fixative. The nasal septum was detached, and the nasal mucosa was removed. Lymph nodes in the neck and abdominal cavity were examined. The excised NALT and nasal mucosa were washed with phosphate‐buffered saline to remove blood. One portion was immediately fixed in 4% paraformaldehyde, whereas another portion was frozen at −80°C. The fixed NALT and nasal mucosa were routinely embedded in paraffin, sectioned, stained with hematoxylin and eosin, and observed under a microscope to assess pathological changes in tissue morphology.

Serum levels of IL‐4, IL‐13, OVA‐sIgE, and ECP, and serum and tissue levels of TNF‐α were measured using enzyme‐linked immunosorbent assay (ELISA) to evaluate the induction of allergy and chronic inflammation. Tissue levels of IL‐17 and TGF‐β were measured using Western blot. Tissue homogenates were centrifuged, and the supernatants were collected. Whole blood samples were centrifuged to obtain serum. ELISA was performed according to the manufacturer's instructions. Arterial blood gas analysis was performed to assess the efficacy of hypoxic induction. Immediately after the final hypoxic stimulation, rats were anesthetized, and the abdominal aorta was punctured using vacuum tubes coated with an anticoagulant. A 1‐mL syringe was used to draw 0.3 mL of blood from the collection tube, avoiding air bubbles. After the first one to three drops were discarded, the blood was gently dispensed into the test card and analyzed using a blood gas analyzer.

#### 2.4.3 Predictive validity

Rats in the treatment group were treated with mometasone furoate and montelukast sodium for 1 week after modeling ended on day 29. Based on a pediatric dosage of 5‐mg montelukast and 50‐μg mometasone furoate in each nostril daily for a 9‐year‐old child,^37^ the doses were converted to rat equivalent doses using body surface area (BSA) calculated using the Stevenson formula for humans, BSA = 0.0061 × height (cm) + 0.0128 × weight (kg) − 0.1529, and the Meeh–Rubner formula for rats: 9.1 × weight (g)2/3/10 000. Montelukast suspension was prepared and administered by oral gavage. Mometasone furoate was administered intranasally dropwise. After treatment, nasal symptoms were observed, and the same tissue collection, histological examinations, and biochemical analyses described earlier were performed to compare with the other groups.

### 2.5 Statistical analysis

Data were presented as mean ± standard deviation ($\bar{X}\pm S$) if normally distributed, and homogeneous variance was assumed. One‐way analysis of variance (ANOVA) and least significant difference *t*‐test were used for multiple comparisons, and independent samples *t*‐test was used for two‐group comparisons. Nonnormally distributed data were expressed as median (*M*) and interquartile range (25th and 75th percentiles) and analyzed using the Kruskal–Wallis H‐test and Nemenyi test for multiple comparisons, and Wilcoxon rank‐sum test for two‐group comparisons. *p* < 0.05 was considered statistically significant. SPSS 26.0 was used for statistical analyses.

## 3 RESULTS

### 3.1 Face validity

#### 3.1.1 Nasal symptoms

**FIGURE 2:**
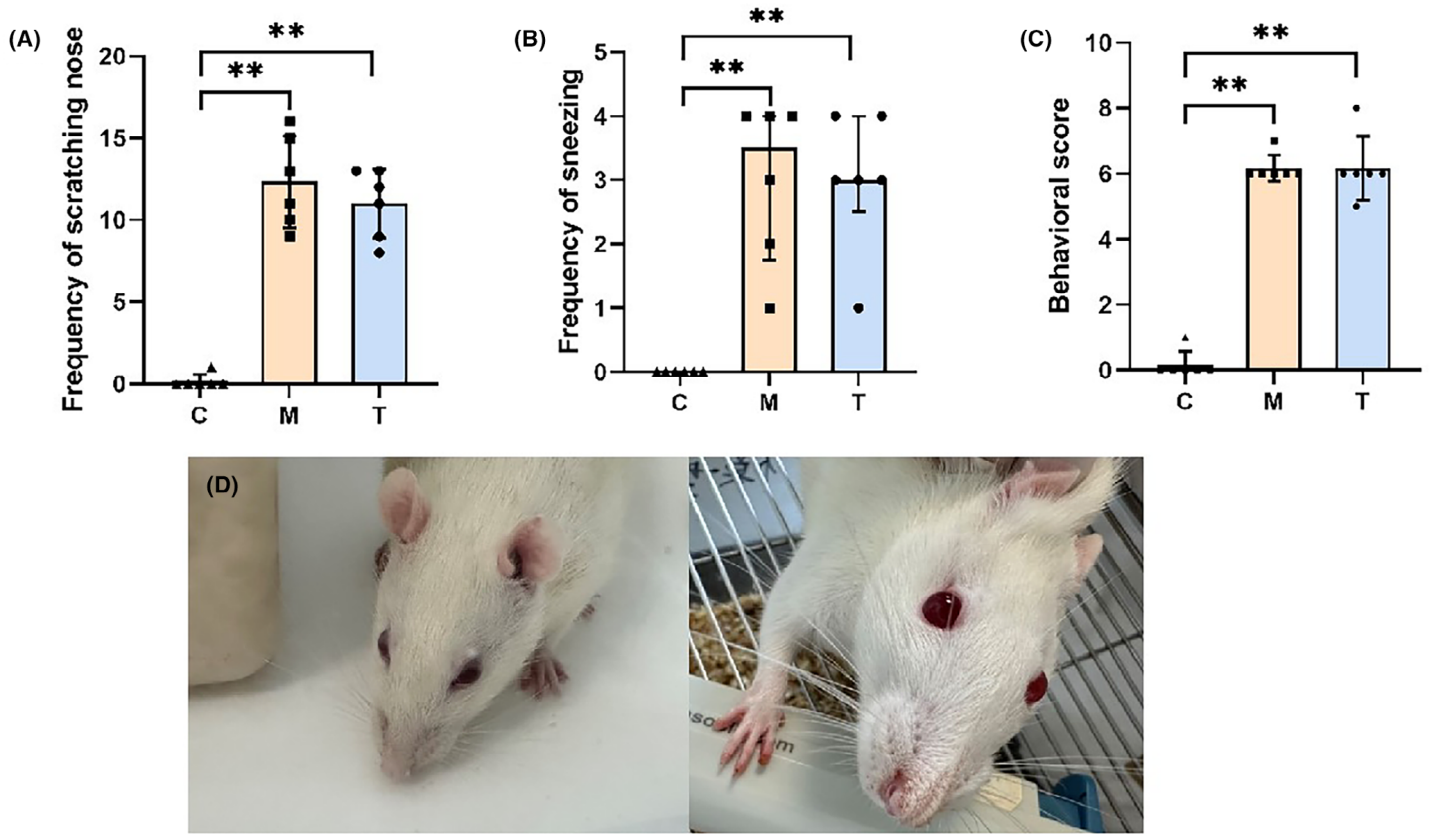
(A–C) The number of nose scratching, sneezing, and overall behavioral scores before treatment in the model and treatment groups was significantly higher than that in the control group (all *p* < 0.01). (D) Cyanosis of the ears, nose, and claws in rats was observed during hypoxia (left), with some rats exhibiting restlessness or drowsiness, showing an increased respiratory rate compared to normoxic rats (right). Both the model and treatment groups exhibited significant nasal and hypoxic symptoms.

Nasal symptoms were scored within 1 h after the final OVA challenge. As the data did not conform to normality, median and interquartile range were used. Numbers of scratches and sneezes were significantly higher in the model [12.00 (9.75,15.25)] and pretreatment [11.50 (8.75,13.00)] groups compared to controls [0.00 (0.00,0.25)]. Sneeze counts showed similar increases in the model [3.50 (1.75,4.00)] and pretreatment [3.00 (2.50,4.00)] groups compared to controls [0.00 (0.00,0.00)]. Rhinorrhea severity scores were also elevated in the model [6.00 (6.00,6.25)] and pretreatment [6.00 (5.75,6.50)] groups compared to controls [0.00 (0.00,0.25)] (Figure 2A–C).

During hypoxic stimulation, cyanosis of ears, nose, and nails was observed in rats along with restlessness, somnolence, and tachypnea compared to normoxia (Figure 2D).

### 3.2 Structural validity

#### 3.2.1 Allergic response

**FIGURE 3:**
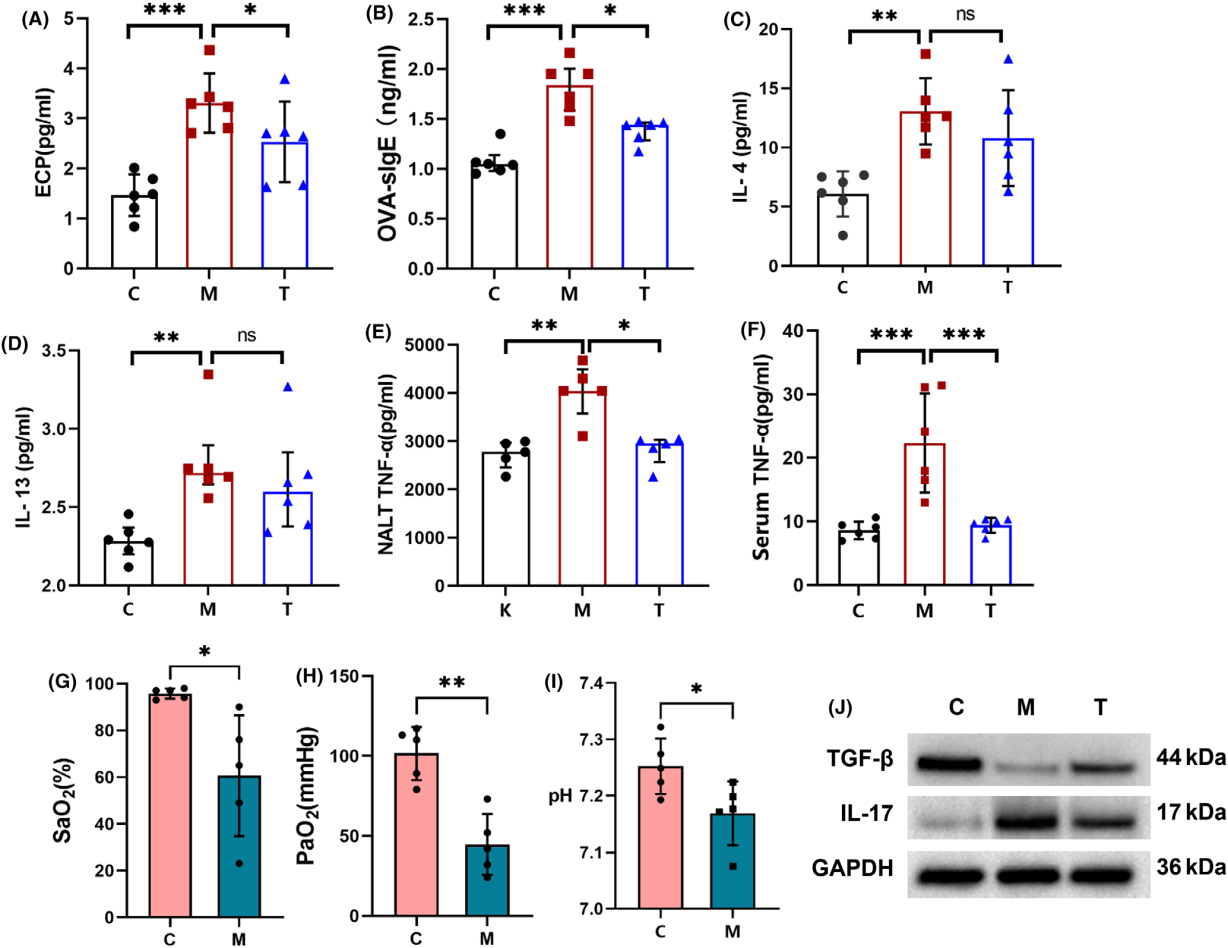
(A–D) Allergen‐related indicators. In the model group, levels of eosinophil cationic protein (ECP), OVA (ovalbumin)‐sIgE, (interleukin) IL‐4, and IL‐13 were significantly elevated compared to the blank control group. After 1 week of treatment, there was evident relief in ECP and OVA‐sIgE levels in the treatment group, and although IL‐4 and IL‐13 levels showed a decline, the differences compared to the model group did not reach statistical significance, possibly due to the short treatment duration. The levels of tumor necrosis factor (TNF‐α) in (E) nasopharynx‐associated lymphoid tissue (NALT) tissue and (F) serum, respectively. Elevated TNF‐α levels were observed in both serum and NALT of the model group, indicating successful induction of chronic nasal inflammation in rats. After 1 week of treatment, TNF‐α levels were markedly alleviated. (G, H, I) Blood gas analysis between the blank control and model groups revealed significant hypoxia in the model group. “ns” denotes nonsignificant differences, *p < 0.05, **p < 0.01, and ***p < 0.001. (J) The levels of IL‐17 and transforming growth factor (TGF)‐β in NALT tissue.

Allergy induction was evaluated by comparing ECP, OVA‐sIgE, IL‐4, and IL‐13 levels between the model and control groups, combined with histological examination. ECP was nearly 10‐fold higher in the model group (10.805 ± 4.045) pg/mL compared to the control group (1.464 ± 0.414) pg/mL (Figure 3A). IgE is associated with type I hypersensitivity reactions and is increased in atopic or hypersensitive individuals.^38^ Thus, we used OVA to induce allergic reactions in rats and then monitored OVA‐sIgE, which was higher in the model group [1.836 (1.585, 2.004)] ng/mL than in controls [1.044 (0.978, 1.065)] ng/mL (Figure 3B). IL‐4 was significantly elevated in the model group (13.057 ± 2.803) pg/mL compared to controls (6.090 ± 1.908) pg/mL. IL‐13 was also higher in the model group [2.719 (2.646, 2.896)] pg/mL than in controls [2.283 (2.199, 2.369)] pg/mL (Figure 3C,D). Western blot results indicate elevated IL‐17 expression and downregulation of TGF‐β in the NALT of the model group rats (Figure 3J).

#### 3.2.2 Chronic inflammation

**FIGURE 4:**
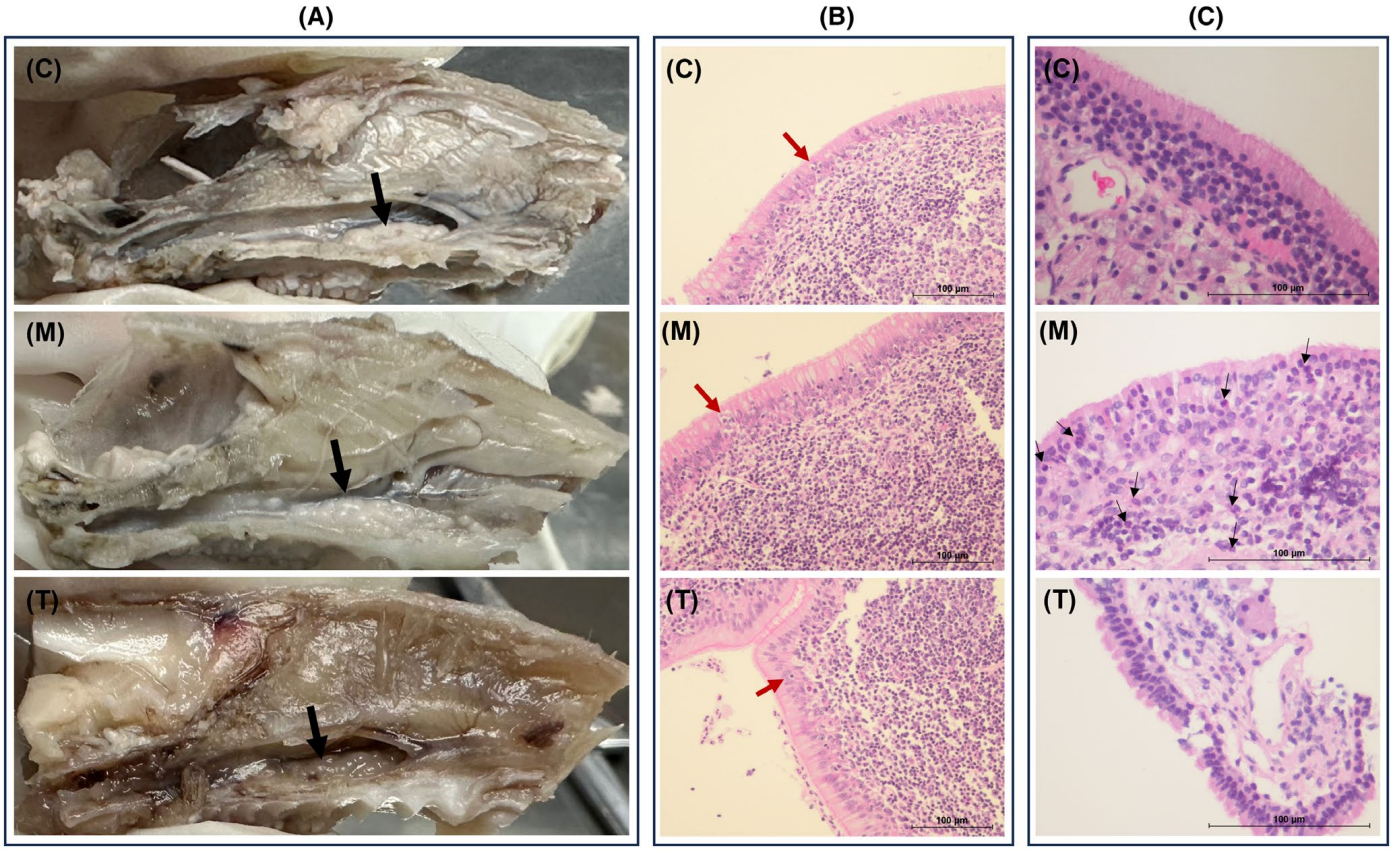
Pathological comparison of rat NALT (nasopharynx‐associated lymphoid tissue) and nasal mucosa tissues. Sections (A) and (B) represent macroscopic and microscopic observations of the nasopharynx‐associated lymphoid tissue (NALT), respectively. Macroscopically, conspicuous lymphoid follicle hyperplasia in the NALT of the model group (indicated by black arrows) was evident, occupying nearly the entire lateral wall of the nasopharynx. This hyperplasia resembled the pathological changes seen in enlarged adenoids and tonsils in humans. Microscopically, sections revealed partial hyperplasia of ciliated epithelium, and the characteristic features of follicle‐associated epithelium (FAE) were observed, with red arrows pointing to M cells (microfold cell) actively phagocytizing other lymphocytes. Posttreatment, NALT size decreased, and follicle hyperplasia alleviated. Section (C) shows the microscopic views of nasal mucosa from the three groups. In the model group, the nasal mucosa exhibited substantial infiltration of eosinophils, neutrophils, and lymphocytes beneath the epithelium, accompanied by interstitial edema. Posttreatment, there was a significant decrease in infiltrating inflammatory cells.

TNF‐α levels and histopathology of the nasal mucosa and NALT were used to evaluate the induction of inflammation. In this study, NALT TNF‐α [4045.792(3574.1214487.584)] pg/mL and serum levels (22.327 ± 7.789) pg/mL were significantly higher in the model group compared to a control group with NALT [2776.102(2455.9642973.441)] pg/mL and serum (8.594 ± 1.382) pg/mL (Figure 3E,F). Histopathology is shown in Figure 4.

#### 3.2.3 Chronic intermittent hypoxia

Arterial blood gas analysis was compared between the control and model groups. The model group exhibited a lower pH (7.169 ± 0.057) than the control group (7.252 ± 0.049). PaO_2_ and SaO_2_ were below normal ranges in the model group, at (44.600 ± 19.047)% and (60.60 ± 25.832)%, respectively. This demonstrates that intermittent hypoxia was successfully induced in the model rats (Figure 3G,H).

### 3.3 Predictive validity

The volume of NALT decreased, with alleviation in follicular hyperplasia, and microscopic analysis reveals minimal inflammatory cell infiltration within the nasal mucosa. Nasal symptoms were compared before and after treatment. Before treatment, the rats exhibited obvious scratching, sneezing, and rhinorrhea, as detailed in Section 3.1.1. After 1 week of treatment, scratching and sneezing decreased to [2.00 (1.00,2.25)]/h and [0.00 (0.00,0.25)]/h, respectively. In addition, behavioral scores were significantly reduced to [1.00 (1.00,2.25)]. Serum OVA‐sIgE [1.440 (1.283,1.464)] ng/mL, ECP (3.305 ± 0.592) pg/mL, serum TNF‐α (9.404 ± 1.835) pg/mL, and NALT TNF‐α [2952.367 (2562.9473030.837)] pg/mL were markedly lower compared to the model group (Figure 3A–F,J). Also, IL‐4 and IL‐13 levels decreased, but differences were not statistically significant compared to the model group (all *p* > 0.05), possibly due to the short treatment duration.

## 4 DISCUSSION

We constructed an AH rat model by simulating the risk factors of AH in this study. The model group exhibited significant nasal symptoms and hypoxia (Figure 2), and NALT lymphoid follicular hyperplasia (Figure 4), which are the most important features of AH. Serum ECP, OVA sigE, IL‐4, IL‐13, and TNF‐α were significantly elevated in the model group, indicating the successful application of allergic and chronic inflammatory stimuli to rats. ECP is an indicator of eosinophil activation and degranulation. M. De Amici found that serum levels of CD163, MPO, and ECP were significantly elevated in children with AH. Therefore, they believed that macrophages may play a relevant pathogenic role as well as neutrophils during bacterial infections and eosinophils in allergic inflammation, ultimately leading to AH.^39^ Abnormalities in cytokines were observed in children with AH. Elevated levels of IL‐4 and IL‐13 are often associated with allergic reactions and are observed in children with AH accompanied by AR.^28^ TNF‐α is often associated with infection or upper‐respiratory‐tract inflammation.^20^ The model group NALT also exhibited Th17/Treg lymphocyte imbalance, which is a clear molecular mechanism of AH in current research.^26^ In general, the model effectively emulates the symptomatic manifestations and pathological alterations characteristic of AH, demonstrating responsiveness to conventional pharmacotherapy.

Notably, the development of an AH animal model has long been a research gap in this field. While various animal models have been devised for OSAHS, they primarily simulate airway obstruction and hypoxia, without adequately elucidating the underlying mechanisms of AH.^40^ Laura D. Serpero et al. developed a mixed cell culture model for the assessment of proliferation in tonsillar tissues. They used tonsils surgically removed from pediatric patients with obstructive sleep apnea and recurrent tonsillitis. Whole tonsillar cells were stimulated using LPS and concanavalin A for 24 h, and cellular proliferation was evaluated using thymidine incorporation.^41^ We previously constructed an AH rat model through the complex induction of AR and chronic pharyngitis.^42^ We did not use the rat NALT analog to adenoids and observed the rat cervical lymph nodes instead, but by that time we had already found similarities between NALT and adenoids.

In this study, we augmented the simulation of CIH and chronic respiratory inflammation based on previous methods, eliminating the use of ammonia‐induced chronic pharyngitis, and analogized the rat NALT to adenoids. Several key considerations were taken into account. First, rats' NALT shares a high structural and functional resemblance with adenoids. Second, given that commonly utilized laboratory animals such as rats and mice lack adenoid tissue, the induction of proliferative hypertrophy to obstruct airways becomes unviable. The limited capacity of AR‐induced nasal mucosal edema hinders the attainment of significant airway obstruction and hypoxia, thereby rendering emulation of the cardinal clinical manifestation of AH‐OSAHS unattainable. Excluding its effects on growth and neural systems, our antecedent fundamental investigations have suggested the involvement of hypoxia in the genesis and progression of localized chronic adenoid inflammation.^33^ Consequently, we assert that incorporating intermittent hypoxia serves as a facsimile of AH symptoms and offers a platform to delve into the mechanisms governing localized adenoid inflammatory processes. Thus, the introduction of hypoxic conditions has been incorporated into the current modeling endeavor. Third, pharyngeal symptoms often result from AH, and not all patients exhibit pharyngeal discomfort and chronic cough. Instead, chronic inflammation resulting from recurrent respiratory infections is the main reason for stimulating AH. Therefore, we excluded the use of ammonia stimulation for chronic pharyngitis and opted to establish chronic nasal inflammation. LPS is a constitutive element of gram‐negative bacterial cell walls that functions as an endotoxin, exerting the potential to simulate facets of the inflammatory cascades elicited by specific infections. Recognized for its utility in creating a spectrum of acute and chronic inflammatory models, including sepsis,^43^ rhinitis,^44^ and chronic pulmonary inflammation,^45^ we have adopted a regimen involving controlled, small‐scale LPS administration via alternate‐day intranasal instillation to faithfully emulate the recurring inflammatory stimuli.

In this paper, we pioneered a method to construct an animal model of AH. However, our observation of NALT, which was used to represent adenoids, was very limited, and only pathologic observation and detection of some inflammatory factors were performed. In the future, we will continue to improve the construction of this model and enhance the observation of NALT by (1) quantifying the degree of hyperplasia and stripping and weighing NALT intact for statistical analysis, (2) observing the alteration in follicle‐associated epithelium, (3) detecting the type and distribution of NALT immune cell types, and (4) comparing these observations with human hypertrophic adenoids to enhance the evaluation of construct validity and further explore the pathogenesis of AH.

## AUTHOR CONTRIBUTIONS

Data curation: Anqi Liu. Funding acquisition: Zhen Xiao. Methodology: Anqi Liu and Zhiyan Jiang. Project administration: Anqi Liu, Yixing Zhang, Yan Lin, Xuejun Li, Shuming Wang, Wenyan Pu, and Xiuxiu Liu. Writing of the original draft: Anqi Liu. Writing—review and editing: Anqi Liu and Zhiyan Jiang. All authors have contributed equally to the manuscript and have read and approved the final version of the manuscript.

## FUNDING INFORMATION

This work was financially supported by the National Natural Science Foundation of China (grant number: 8217150152), the Clinical Science and Technology Innovation Project of Shanghai Shenkang Hospital Development Center (grant number: SHDC12021102), and the Shanghai Three‐Year Action Plan to Further Accelerate the Development of Traditional Chinese Medicine Inheritance and Innovation (grant number: ZY(2021‐2023)‐0209‐05).

## CONFLICT OF INTEREST STATEMENT

The authors declare that there is no conflict of interest with any financial organization regarding the content and details assumed in this paper.

## ETHICS STATEMENT

The experiment and animal handling procedures were approved by the Research Ethics Committee of Shanghai University of Medicine & Health Sciences (2021‐GZR‐18‐340406198707142817).
